# Treatment of dental plaque biofilms using photodynamic therapy: a randomised controlled study

**DOI:** 10.1007/s40368-021-00637-y

**Published:** 2021-06-05

**Authors:** A. Alsaif, J. F. Tahmassebi, S. R. Wood

**Affiliations:** 1 Department of Paediatric Dentistry, University of Leeds, Leeds, UK; 2 Department Oral Biology, University of Leeds, Leeds, UK; 3 Paediatric Dentistry Department, Ministry of Health, Kuwait city, Kuwait

**Keywords:** *In vivo* biofilm, CLSM, Erythrosine, Incubation time, Irradiation time, Antimicrobial

## Abstract

**Introduction:**

Photodynamic therapy (PDT) is a treatment modality involving a dye that is activated by exposure to light of a specific wavelength in the presence of oxygen to form oxygen species causing localised damage to microorganisms.

**Aim:**

To determine the most effective bactericidal incubation and irradiation times of erythrosine-based PDT on *in vivo*-formed dental plaque biofilms.

**Methods:**

A randomised controlled study; 18-healthy adult participants wearing intraoral appliances with human enamel slabs to collect dental plaque samples in two separate periods of two weeks each for use in arm-1 and arm-2. These accumulated dental plaque samples were treated with PDT under different experimental conditions. Incubation times with photosensitiser (erythrosine) of 15 min and 2 min were used in arm-1 and arm-2, respectively, followed by light irradiation for either 15 min (continuous) or as a fractionated dose (5 × 30 sec). Following treatment, percentage reductions of total bacterial counts were compared between the different groups. In addition, confocal laser scanning microscopy (CLSM) and LIVE/DEAD® BacLight™ Bacterial Viability Kit were used to visualise the effect of PDT on *in vivo*-formed biofilms.

**Results:**

Significant reductions in the percentage of total bacterial counts (~93–95%) of *in vivo*-formed biofilms were found when using either 2 min or 15min incubation times and applying 15 min continuous light. Although when applying fractionated light, there was more cell death when 15 min incubation time was used (~ 91%) compared with the 2 min incubation time (~ 64%). CLSM results supported these findings.

**Conclusion:**

Improving the clinical usefulness of PDT by reducing its overall treatment time seems to be promising and effective in killing *in vivo*-formed dental plaque biofilms.

## Introduction

Increases in the incidence of antibiotic/antimicrobial drug-resistant bacteria necessitates the development of alternative approaches to the control of dental plaque (Wainwright and Crossley 2004; Meisel and Kocher 2005). In addition, there are limitations associated with the conventional approaches to controlling dental plaque, such as parents’ lack of awareness/skills of correct brushing technique or difficulty manipulating a brush in the mouth (Marshman et al. 2016). Moreover, it may be exacerbated in individuals with, for example, physical or learning disabilities (Ciancio 1988; Baker 1993) and younger children (Tahmassebi et al. 2015). One such alternative treatment is photodynamic therapy (PDT). PDT involves the application of visible light; a photosensitiser; and oxygen (Takasaki et al. 2009). The photosensitiser binds to the target cells and can be activated by light of a wavelength that corresponds to one of its absorption maxima. Following activation, reactive-oxygen species are produced that are toxic to microorganisms (Soukos and Goodson 2011). PDT possesses several advantages including effective targeting of the treatment by appropriate dosimetry and application of the photosensitiser and light (Hamblin and Hasan 2004; Gursoy et al. 2013). When applied in this way, it provides a noninvasive, confined bacterial killing without harming host tissues and can be carried out in outpatient settings (Konopka and Goslinski 2007; Gursoy et al. 2013).

However, in the previous studies, the PDT treatment time has been longer than would be ideal for the clinical setting (Metcalf et al. 2006; Wood et al. 2006; Tahmassebi et al. 2015). To our knowledge, there were no reports in the literature that have compared the efficacy of PDT on *in vivo* formed plaque biofilms with different incubation times with erythrosine photosensitiser prior to irradiation. Therefore, to further study this and enhance clinical usefulness, this work was conducted aiming to reduce the overall treatment time for PDT by investigating the bactericidal efficacy after different incubation and irradiation times.

## Materials and methods

The principal investigator was trained and calibrated in the use of aseptic microbiological procedures in microorganisms’ cultures as well as in the preparation of study solutions and dilution series and making Columbia Blood Agar (CBA) plates, counting bacterial colonies, using microscopy and CLSM. Also, the principal investigator was trained in the preparation of enamel slabs and making *in situ* appliances prior to commencement of the study and prior the usage of these facilities or materials.

### Photosensitiser and light source

The photosensitiser, erythrosine-B (Sigma-Aldrich, Germany), was prepared as a filter-sterilised 1mg/ml stock solution in Reduced Transport Fluid (RTF) and stored in the dark at 4 °C. It was then diluted to the required 220 μM concentration, which was found by Tahmassebi et al. (2015) to be the most effective bactericidal concentration for PDT on *in vivo-*formed oral biofilms. This was used for all PDT treated groups in this study.

A 400 W-Tungsten filament lamp (Aurora, USA), white light source, was used to activate erythrosine. It emitted 22.7 mW/cm^2^ of light intensity in the wavelength range 500–550 nm (the region of maximal absorption by erythrosine). Bacterial samples were placed at 30 cm from the lamp and the heat-dissipating water bath (Stuart, SBS40, UK) was positioned between the samples and the lamp to prevent heating.

### In situ appliance

**Fig. 1:**
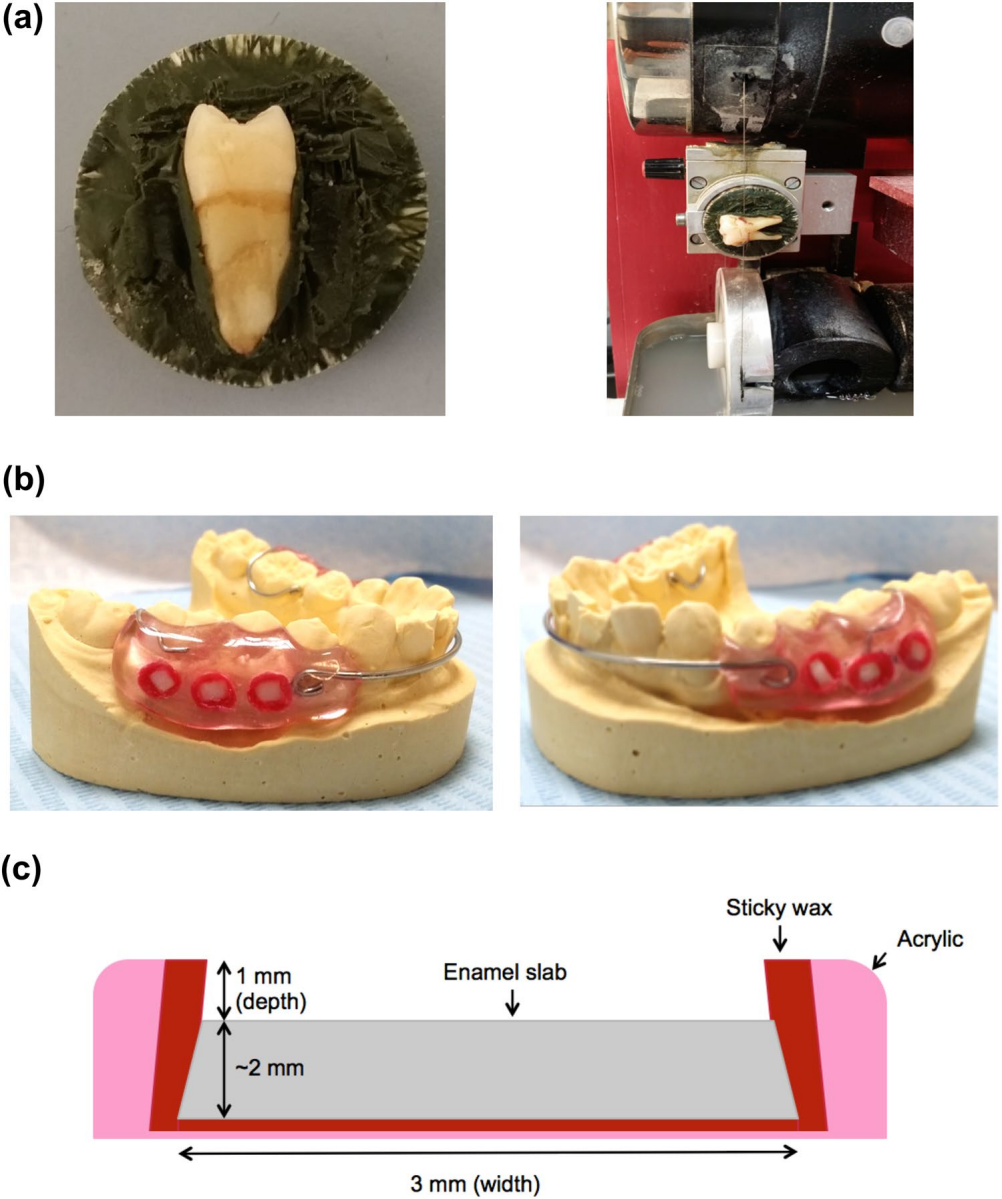
Preparation of *in situ* appliance. **a** Illustration of tooth sectioning. **b** Right and left lateral views of *in situ* appliance. **c** Enamel slab in *in situ* appliance

Mandibular removable appliances were fabricated according to a previously published design (Tahmassebi et al. 2015). The appliances consisted of a labial arch wire, acrylic flanges buccally on premolars and first permanent molars and a U clasp attached to each of the first permanent molars. Three sterilised human enamel slabs (3 mm × 2 mm × 2 mm) were inserted in the right buccal flange and another three in the left buccal flange of each appliance, leaving a 1 mm depth for plaque accumulation on the surface of the slab (shown in Fig. 1).

The enamel slabs were cut from sound human premolars and permanent molars extracted for orthodontic or periodontal reasons at the School of Dentistry using a water cooled, Diamond Wire Saw, cutting machine (Well® Walter EBNER, CH-2400 Le Loche, Germany). These teeth were stored in a solution of distilled water and 0.1% thymol (Sigma Aldrich, Germany) at room temperature.

Prepared enamel slabs were kept moist in distilled water with thymol. Later, they were immersed overnight in sodium hypochlorite (12%), followed by thorough rinsing with deionised water and immersed overnight in phosphate buffered saline (pH 7.4) and sent to the Department of Immunology of the University of Liverpool, where they were exposed to gamma radiation (4080Gy). This level of exposure provides sterilisation without altering the structural integrity of the enamel slabs (Amaechi et al. 1999). In addition, the use of hypochlorite to treat enamel does not affect biofilm development (Watson et al. 2004). Enamel slabs were sterilised twice prior to each arm of the study.

### Ethical aspects

Ethical approval was obtained from the National Research Ethics Service committee of South-Central Berkshire-B (REC reference number: 14/SC/1226) and the Leeds Research and Innovation Committee (DT14/11310 (149271/WY)). A summary of this study was registered online in a publicly accessible database (NHS Health Research Authority) before subject recruitment. This study was conducted in full conformance with the laws and regulations of the country in which the research was conducted and as per the World Medical Association Declaration of Helsinki.

### Study design and participants

This was a single centre, randomised study with two arms design. Eighteen-healthy participants (16 females, 2 males), with mean age 34 years (± 10.27), were recruited at the Leeds School of Dentistry. The sample size was calculated using the data from the previous pilot study (Tahmassebi et al. 2015) using a power calculation 80%, which resulted in a sample size of at least five per treatment group. An explanatory information sheet was provided to each participant. Participants had to meet the inclusion and exclusion criteria for the study. The inclusion criteria were medically fit, normal salivary flow rate (≥ 0.25 ml/min), DMFT ≥ 1 and to fully understand the procedures and restrictions and willing and likely to comply, as evidenced by voluntary written informed consent. Exclusion criteria included active oral disease, use of antimicrobial therapy and/or antibiotics within 14 and 28 days prior to screening or during the study, use of medication affecting salivary flow and wearing of prostheses that could affect the study procedures. The participants were instructed to wear custom-made appliances, at all times for two separate periods of two weeks each, except when eating, drinking and tooth brushing. At these times, participants placed the appliance in damp gauze inside a provided plastic case (Henry Schein, USA), to prevent any drying of the accumulated plaque. The enamel slabs were coded and randomly allocated to the *in situ* appliances using a random number generator (http://stattrek.com/statistics/random-number-generator).

### PDT on *in vivo* formed plaque biofilms

After 14 days of wearing the appliances, the enamel slabs with the accumulated biofilms were removed from the appliances and were randomly allocated to the different groups using sealed envelopes. The samples were treated under four experimental conditions; (a) control 1 (no erythrosine, no light) represented by 1-slab; (b) control 2 (erythrosine, no light) represented by 1-slab; (c) treatment-1 (erythrosine, +15min continuous light) represented by 2-slabs (T1A and T1B); (d) treatment-2 (erythrosine, +30 sec light pulses for 5-times, separated by dark periods of 1min) represented by 2-slabs (T2A and T2B). Duplicate slabs were treated in treatment 1 and 2 groups to assess the variation of bacterial viability values between the duplicates of the same group with a view to validate and increase the reliability of the results.

Samples from 15 participants were used for the microbiological killing assay to determine the bacterial viability values between the different groups. The samples from the remaining 3 participants were used for viewing under CLSM.

### Arm 1 and 2

**Fig. 2:**
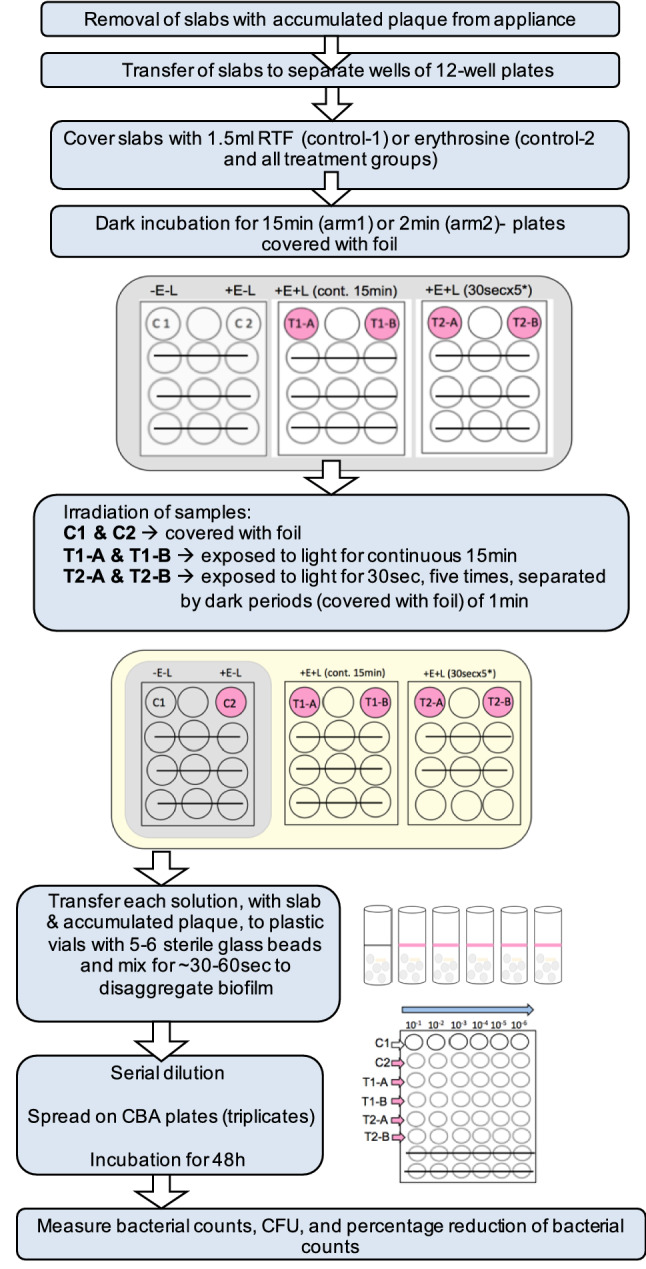
Laboratory protocol of PDT on *in vivo*-formed plaque biofilms

Figure 2 shows the experimental conditions and the laboratory protocol regime which were the same for the two arms of the study, except that the incubation time of the plaque biofilms with photosensitiser was different. In the 1st arm, the incubation time was 15 min, a duration of time that was used in the previous study, Tahmassebi et al. (2015), whereas, in the 2nd arm, the incubation time was 2 min, a duration of time that was found, by our team, to be as effective as the 15 min incubation time in bacterial killing of planktonic cultures of *L. casei* spp. (un-published data)*.* In brief, the six slabs, with accumulated plaque, were removed from the appliance and individually transferred to separate wells of 12-well tissue culture plates (Costar, Sigma-Aldrich, Germany) and completely covered with either 1.5 ml of RTF (C1) or 1.5 ml (220 μM) erythrosine (C2, T1A, T1B, T2A and T2B). Next, the 12-well plates were covered with foil and incubated for 15 min (Arm-1) or 2 min (Arm-2) at room temperature. After dark incubation, foil was removed from the wells and all plates were placed under the lamp. The treatment groups (T1A and T1B) were irradiated continuously for 15 min, while treatment groups (T2A and T2B) were irradiated with 30 sec light pulses (×5), separated by dark periods of 1 min, using foil to cover these wells during the dark periods. Control groups (C1 and C2) were placed under the lamp covered with foil (erythrosine, no light); in an attempt to treat all groups under same conditions as far possible. The bacterial solutions from each well, with the enamel slab and the accumulated plaque, were then individually transferred to sterile plastic vials (Sterilin, UK), and mixed for ~ 30–60 sec, using vortex mixer (Genie-2, scientific industries, Inc., USA), with 5–6 sterile glass beads of 3 mm diameter each (Sigma-Aldrich, Germany), to disaggregate the plaque biofilms. To determine the number of bacteria in a sample, tenfold serial dilutions were prepared from the bacterial suspensions of the control and treatment groups. 100 μl of each dilution was then plated on Colombia blood agar (CBA) (Oxoid, Basingstoke, UK) in triplicate (Total = 108 plates for each appliance). The plates were then incubated at 37 °C in a 10% CO_2_ incubator (Forma Direct Heat, Thermo Scientific, USA) for 48 h. After incubation, bacterial counts of each plate were quantified using a colony counter (Stuart R, SC6, UK) and the colony-forming units per millilitre (CFU/ml) was calculated from the mean count of each triplicate.

### CLSM observations

To visualise the effect of PDT on the *in vivo* formed biofilm, samples from 3-participants were treated under different conditions; (a) control (no erythrosine, no light); (b) treatment-1 (erythrosine, +15 min continuous light); (c) treatment-2 (erythrosine, +30 sec light pulses for 5-times, separated by dark periods of 1min). Following treatment, samples were stained using the LIVE/DEAD® BacLight™ Bacterial Viability Kit (L7012) (Molecular Probes, Eugene, OR, USA) as per the manufacturer’s instructions. The biofilm samples were stained with SYTO9 and Propidium iodide for 15 min in the dark. The labelled biofilms were viewed using CLSM (Leica TCS SP2, Wetzlar, Germany), to investigate, *in situ*, the viability of bacteria within the biofilm. Samples were examined using excitation with an argon (488 nm) and HeNe (543 nm) lasers with ×10 and ×63 water-dipping objective lenses.

### Statistical analysis

Microbial counts were expressed as the number of colony-forming units (CFU) per ml. Each CFU value represented the mean count from triplicate samples. Logarithmic transformation (log_10_) was applied to the original observations (CFU/ml) to make the distribution more symmetric. Percentage reduction in bacterial counts in each group was calculated by dividing the difference in CFU between control and treatment groups with the number of CFU from the control group from the same subject.

Tests were performed using IBM**®** SPSS**®** Statistics Version23. A significance level of α < 0.05 was implemented. The parametric ANOVA test was used for the normally distributed continuous data and the nonparametric Friedman test was used for the skewed continuous data, followed by a series of Wilcoxon tests to identify where the specific difference lay with adjustments for multiple comparisons (α = 0.05/number of comparisons). Bland–Altman plot was used to evaluate the Intra-examiner reproducibility. Bacterial counts of 10% of the total number of CBA plates used in both arms were randomly re-counted 5–7 days following the initial counting.

## Results

To ensure that the baseline measurement of the accumulated plaque in all the six slabs were comparable, we investigated the variation of bacterial viability counts among these slabs from one participant prior to the main study PDT investigation. It revealed no variation in the CFU/ml among these six *in vivo* formed biofilm samples over the 14 days of plaque accumulation (Coefficient of variation = 0.01). This was in agreement with the previous studies carried out by our team (un-published data) and Arweiler et al. (2004).

### Arm-1: 15 min incubation time

**Fig. 3:**
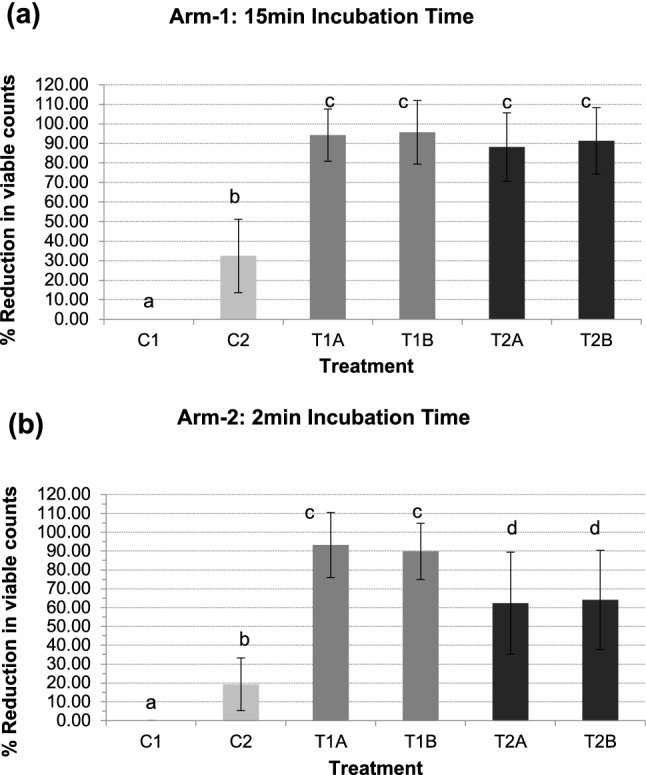
Percentage (%) reduction in viable counts of *in vivo* formed biofilms under different experimental conditions: C1 (No erythrosine, no light); C2 (Erythrosine, no light); T1A and T1B (Erythrosine, +15 min continuous light); T2A and T2B (Erythrosine, +30 sec light pulses for 5 times, separated by dark periods of 1 min) (**a**, **b**). Data represent median values **a** (*n *= 13) **b** (*n *= 15). Error bars represent SD and data followed by different letters differ statistically (*p* < 0.003).

The percentage reduction in viable counts following each irradiation protocol is shown in Table 1a and Figure 3a. The results showed a statistically significant difference in the percentage reduction of bacterial counts between the two control groups (*p* = 0.001) and also between these control groups with the four treatment groups in all subjects, *p *= 0.001 (< 0.003). All four treatment groups had a significantly higher percentage reduction in their bacterial counts than the control groups. However, there was no statistically significant difference between the four treatment groups (*p* > 0.003).

**Table 1:** Percentage (%) reduction in viable counts and mean log10 CFU/ml obtained from the *in vivo* formed biofilms treated under different experimental conditions when using an incubation time of 15 min (**a**) or 2 min (**b**): C1 (No erythrosine, no light); C2 (Erythrosine, no light); T1A and T1B (Erythrosine, +15 min continuous light); T2A and T2B (Erythrosine, +30 sec light pulses for 5 times, separated by dark periods of 1 min)

(a) Arm-1: 15 min incubation time						
Treatment conditions	*N*	Mean % (SD)	Min. %	Max. %	Median %	Mean log10 CFU/ml (SD)
(C1): E−L−	13	0.00 (±0.00)	0.00	0.00	0.00	6.08 (±0.79)
(C2): E+L−	13	42.14 (±18.76)	17.08	74.66	32.46	5.82 (±0.76)
(T1A): E+(15 min)L+	13	89.13 (±13.35)	64.03	99.82	94.27	4.72 (±0.80)
(T1B): E+(15 min)L+	13	87.45 (±16.33)	55.40	99.68	95.73	4.75 (±0.81)
(T2A): E+(5 × 30 s)L+	13	83.15 (±17.50)	46.39	99.47	88.15	5.06 (±0.86)
(T2B): E+(5 × 30 s)L+	13	85.10 (±16.92)	47.48	99.71	91.37	4.95 (±0.90)

(b) Arm-2: 2 min incubation time						
Treatment conditions	*N*	Mean % (SD)	Min. %	Max. %	Median %	Mean log10 CFU/ml (SD)
(C1): E−L−	15	0.00 (±0.00)	0.00	0.00	0.00	5.76 (±0.72)
(C2): E+L−	15	20.21 (±13.99)	3.31	46.13	19.26	5.66 (±0.70)
(T1A): E+(15 min)L+	15	85.05 (±17.25)	52.31	99.30	93.22	4.52 (±0.72)
(T1B): E+(15 min)L+	15	85.75 (±14.92)	56.52	99.35	89.92	4.62 (±0.65)
(T2A): E+(5 × 30s)L+	15	63.50 (±27.10)	25.41	98.89	62.34	5.13 (±0.64)
(T2B): E+(5 × 30s)L+	15	68.77 (±26.32)	18.86	99.20	64.09	4.95 (±0.83)

### Arm-2: 2 min incubation time

The percentage reduction in viable counts following each irradiation protocol is shown in Table 1b and Figure 3b. The results showed a statistically significant difference in the percentage reduction of bacterial counts between the two control groups (*p *= 0.001) and also between these control groups with the four treatment groups in all subjects, *p *= 0.001 (< 0.003). All four treatment groups had a significantly higher percentage reduction in their bacterial counts than the control groups. In addition, there was a statistically significant difference between the treatment groups irradiated with 15min continuous light (T1A and T1B) and the groups with the fractionated light (*p* < 0.003) with a higher percentage reduction of bacterial counts (up to ≈ 93%) seen in the 15 min continuous light groups (T1A and T1B).

In terms of intra-examiner reliability, one sample *t* test showed a nonstatistically significant difference between the counts performed in two different time periods (*p *= 0.220, > 0.05). Furthermore, a Bland–Altman plot showed a high level of agreement between the two measurements (most of the values were close to the zero line and within the 95% confidence interval limits).

### CLSM observations

**Fig. 4:**
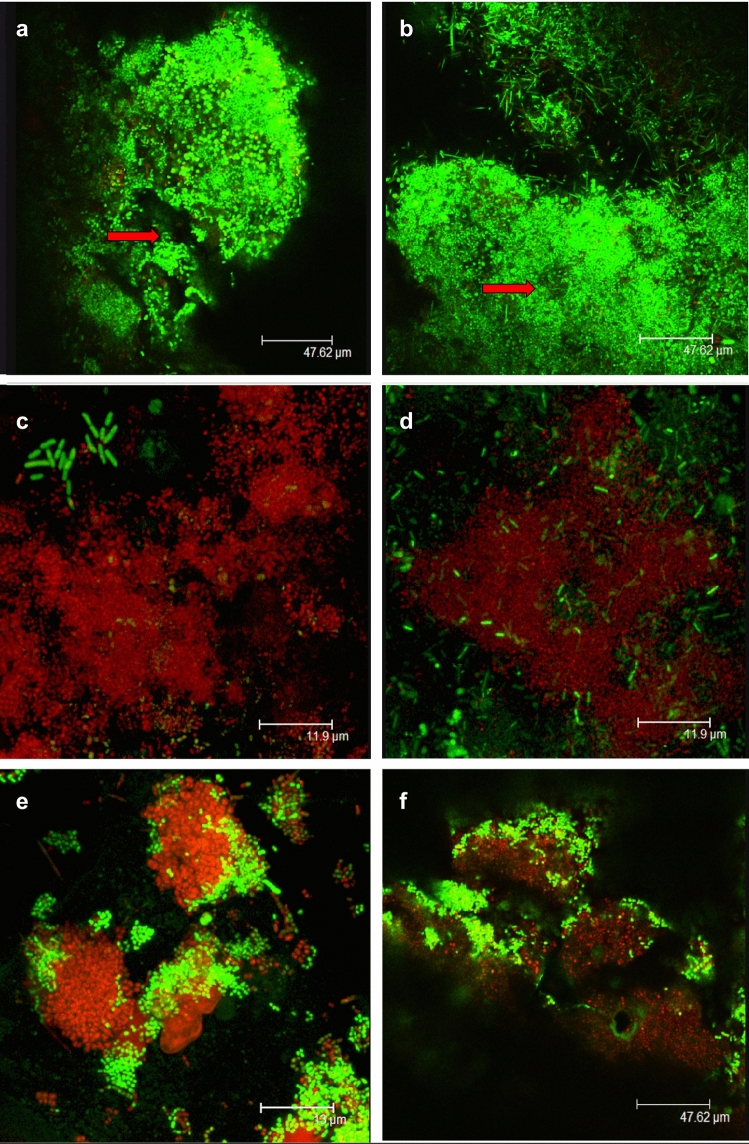
CLSM images of 14-day *in vivo* formed biofilm samples (**a**–**f**). **a**, **b** Untreated (control) biofilm samples. Aggregates of bacteria were separated by fluid filled voids (black holes-red arrow). **c**, **d** Biofilm samples incubated with erythrosine (220 μM) for (**c**) 15 min or (**d**) 2 min and irradiated for continuous 15 min. **e**, **f** biofilm samples incubated with erythrosine (220 μM) for (**e**) 15 min or (**f**) 2 min and irradiated for fractionated 5 × 30 sec with 1min dark recovery periods. Green fluorescence indicates viable bacteria and red/yellow fluorescence indicates affected bacteria. All images were taken with ×63 lens

Figure 4 shows CLSM images of PDT-treated and control biofilms. The majority of the cells showed green fluorescence in the absence of irradiation and erythrosine, indicating a high level of cell viability. However, biofilm samples treated with PDT showed an increase in red/yellow fluorescence, indicative of dead cells.

## Discussion

The present study investigated the bactericidal effect following 15 min continuous irradiation and, also, following a fractionated regime of 30 sec light pulses for 5 times separated by 1 min dark periods in an attempt to reduce the overall PDT treatment time to enhance its clinical usefulness.

Interestingly, the data from the present study showed that when the irradiation time was reduced to 2.5 min, which was applied in 30 sec light pulses for 5-times separated by 1min dark periods, the bacterial killing (~ 91%) was effective and equal to bacterial killing obtained from the 15 min continuous irradiation (~ 95%) when 15 min incubation time (Arm-1) with erythrosine was used. This may be due to the replenishment of oxygen during the dark periods for erythrosine to initiate more photochemical reactions or perhaps due to the replenishment/redistribution of the erythrosine itself within the biofilm during these dark periods as continuous irradiation may lower erythrosine levels due to photo bleaching (Metcalf et al. 2006; Wood et al. 2006).

These findings agree with Tahmassebi et al. (2015), where their results suggested that the bactericidal effect of PDT was photosensitiser and light dose-dependent (Konopka and Goslinski 2007; Tahmassebi et al. 2015). However, the bacterial reductions observed in the previous studies on *S. mutans* biofilms grown *in vitro* were higher (3–3.7 log_10_) (Metcalf et al. 2006; Wood et al. 2006) than those found in the current study on *in vivo*-formed biofilms (1–1.36 log_10_). A potential explanation is that *in vitro*-formed biofilms have a limited number of species and have a composition and structure that is different to those in *in vivo*-formed biofilms (Watson et al. 2005). As a result, the analysis of undisturbed human dental plaque biofilms has been considered as the best method for studying the effect of antimicrobial therapies on the biofilm structure (Tomás et al. 2010).

The effectiveness of antimicrobial PDT has also been reported to be dependent on the incubation time of micro-organisms with photosensitiser (Andrade et al. 2013). Various erythrosine-mediated PDT studies have used different incubation times prior to irradiation (Metcalf et al. 2006; Wood et al. 2006; Chibebe Junior et al. 2010; Rolim et al. 2012; Pereira et al. 2013; Tahmassebi et al. 2015). However, there were no reports in the literature that have compared the efficacy of different incubation times on *in vivo*-formed biofilms.

In the present study when 2 min incubation time was used, there was less cell death (64%) when applying fractionated light compared to the 15 min continuous light (~ 93%). Some erythrosine-based PDT studies have used 5min incubation time (Chibebe Junior et al. 2010; Rolim et al. 2012; Pereira et al. 2013), while others have used 15 min incubation time (Metcalf et al. 2006; Wood et al. 2006; Tahmassebi et al. 2015) and both times showed significant reduction in the bacterial viability. The reasons for this difference in the present study are unclear, but may be due to the shorter contact time with the photosensitiser that could have limited its uptake into the bacterial cells, decreasing (but not negating) the PDT effect.

This outcome partly contradicted the results found in our initial PDT study on planktonic cultures of *L.casei*, where different incubation times (2, 5 and 15 min) presented no statistical differences on the percentage reduction of bacterial counts (un-published data). This suggests that the effect of PDT on dental biofilms is different than on planktonic cells. This is likely due to the structural variation in the biofilms and in bacterial cell membranes or the presence of other components, such as extracellular matrix and quorum-sensing factors in dental biofilms, which render the biofilm bacteria more resistant to treatment than planktonic cultures (Huang et al. 2012).

CLSM was used to visualise PDT effects on *in vivo*-formed biofilms. The present analyses agreed with the previous studies’ findings on biofilm structure as it showed a heterogeneous architecture in terms of types of cells present, such as cocci, rods and filaments and also in terms of the overall structure where clumps of bacteria were surrounded by extracellular matrix and separated by voids/channels (black holes) (Wood et al. 2000; Lawrence et al. 2003; Dige et al. 2007). These voids were thought to be filled with biological substances, such as extracellular polymeric substances (EPS) and glycoproteins and act as a circulatory system where oxygen and nutrients were available in the biofilm (Wood et al. 2000). The few previous studies that have evaluated the antibacterial effect of PDT using erythrosine by CLSM analysis have used *in vitro*-formed *S. mutans* biofilms (Wood et al. 2006; Lee et al. 2013). In the present investigation, CLSM images showed a marked difference in the bacterial viability between the control and the PDT-treated samples. In the control samples, almost all cells were stained green, indicating viable bacteria. In the treated samples, there was uneven spatial distribution of vital and dead cells with a higher proportion of dead cells. These findings corresponded with the findings of Lee et al. (2013) when PDT effects was investigated against *in vitro*-formed biofilms using erythrosine and a dental halogen curing light. However, in the present investigation, it was not possible to determine which treatment group was superior in terms of bacterial killing as no quantitative technique was performed. Nevertheless, there are several explanations for the uneven distribution of these vital and dead cells throughout the biofilms such as the availability of oxygen for PDT to have an effect or having diverse populations of cells that have different susceptibilities to PDT. Interestingly, this was seen in some plaque samples (shown in Fig. 4c) where the rod-shaped bacteria seem to be less susceptible to PDT than the coccoid bacteria.

Overall, the bactericidal effects of PDT as seen by CLSM has corresponded with the findings of this present study when using the microbiological analysis of viable bacterial count, indicating that a combination of microbiological techniques and microscopic techniques can help to achieve a realistic representation of PDT effect on *in vivo* formed dental plaque biofilms.

Evidently erythrosine-based PDT can cause significant reduction of *in vivo* formed plaque biofilms and might be useful for controlling dental plaque related diseases such as caries and periodontal disease. Therefore, well conducted *in situ* studies/trials are required to assess the effect of PDT clinically if they exist and compare it with the current best practice. However, prior to the transition to clinical trials, the mode of delivery of PDT in oral cavity needs to be established.

Although the tungsten filament lamp is effective for erythrosine-based PDT, the drawback of this light source is that it is bulky and, also, generates heat that might cause a burning sensation *in vivo* if not appropriately dissipated. Therefore, this source might not be convenient for clinical use in patients. One possible alternative would be to use an LED light source. These have already been used in dentistry as a curing light for restorative materials (Rueggeberg et al. 2017) and are readily available, small, portable and safe. In addition, LEDs do not generate heat like the tungsten filament lamp. Several antimicrobial erythrosine-based PDT studies have used LEDs and showed successful outcomes (Chibebe Junior et al. 2010; Rolim et al. 2012; Pereira et al. 2013).

In addition, erythrosine has already been used in dentistry to stain and visualise dental plaque in the form of disclosing solution or tablets to facilitate oral hygiene instructions (Wood et al. 2006). In plaque disclosing solutions, it is used at concentrations of 9–25 mM (0.72–2% weight/volume) (Marsh et al. 1989), which is much higher than that used in several PDT studies (Tahmassebi et al. 2015; Metcalf et al. 2006; Wood et al. 2006) and, also, in this current study, which was 220 μM. Erythrosine’s antimicrobial property against Gram-positive and Gram-negative oral bacteria (Marsh et al. 1989; Baab et al. 1983; Caldwell and Hunt 1969; Begue et al. 1966) as well as its light-absorbing property and the ability to initiate photochemical reactions (Wood et al. 2006) are well documented in the literature. Therefore, erythrosine presents advantages over other dyes for the use in PDT, because it is already been approved for the use in dentistry by the US Food and Drug Administration (FDA) to visualise dental plaque. In addition, it does not show direct toxicity to the host tissue (Carrera et al. 2016; Ganesan et al. 2011; Allaker and Douglas 2009; Wood et al. 2006; Arnim 1963).

Therefore, the combination of an LED light source and erythrosine varnish should be considered and studied for PDT clinical trials in the near future. As both of them are commonly used in dental clinics, PDT could be made available without significant additional cost. However, its cost effectiveness should be measured against the current best practice.

This technique can be used at home as an adjunct to mechanical removal or by itself where brushing is not tolerated, such as in cases with mucositis, to decrease the oral bacterial load; hence, preventing/controlling dental caries and periodontal disease. It can be delivered in a way similar to the bleaching trays with attachable LED light unit and integrated timer. The erythrosine can be supplied as a varnish or in injectable syringes for ease of topical application. However, more research is needed to assess its effectiveness compared with the current best practice.

## Conclusions

This study has shown that improving the clinical usefulness of photodynamic therapy is possible by reducing the incubation time with erythrosine from 15 min to 2 min with 15 min irradiation time. Further research is; however, needed to investigate a more clinically acceptable irradiation time, as this is particularly necessary for special needs patients and younger children with short attention span.
